# Hepatitis E Pathogenesis

**DOI:** 10.3390/v8080212

**Published:** 2016-08-05

**Authors:** Sébastien Lhomme, Olivier Marion, Florence Abravanel, Sabine Chapuy-Regaud, Nassim Kamar, Jacques Izopet

**Affiliations:** 1 INSERM, UMR1043, Department of Virology, CHU Purpan, Université Paul Sabatier, 31000 Toulouse, France; lhomme.s@chu-toulouse.fr (S.L.); marion-olivier@hotmail.fr (O.M.); abravanel.f@chu-toulouse.fr (F.A.); chapuy-regaud.s@chu-toulouse.fr (S.C.-R.); 2 INSERM, UMR1043, Department of Nephrology, Dialysis and Organ Transplantation, CHU Rangueil, Université Paul Sabatier, 31000 Toulouse, France; kamar.n@chu-toulouse.fr

**Keywords:** hepatitis E virus, pathogenesis, extra-hepatic manifestation, fulminant hepatitis, chronic infection

## Abstract

Although most hepatitis E virus (HEV) infections are asymptomatic, some can be severe, causing fulminant hepatitis and extra-hepatic manifestations, including neurological and kidney injuries. Chronic HEV infections may also occur in immunocompromised patients. This review describes how our understanding of the pathogenesis of HEV infection has progressed in recent years.

## 1. Introduction

The hepatitis E virus (HEV) is the major cause of viral hepatitis. It is distributed worldwide and has been responsible for outbreaks in developing countries and sporadic cases in both developing and developed countries [1]. HEV is mainly transmitted enterically. HEV genotype 1 (HEV1) and genotype 2 (HEV2) are prevalent in developing countries and are found only in humans. Other HEV genotypes, including genotype 3 (HEV3) and genotype 4 (HEV4), have been detected in both humans and animals, with pigs representing a major reservoir. While most infections are asymptomatic, HEV causes acute hepatitis which can be severe in patients with pre-existing liver disease and in pregnant women in developing countries. These infections may have extra-hepatic manifestations and may lead to chronic hepatitis in immunocompromised patients. The pathogenesis is still unclear but recent studies with animal models and cell culture systems will undoubtedly improve our knowledge.

## 2. HEV and Classification

**Figure 1:**
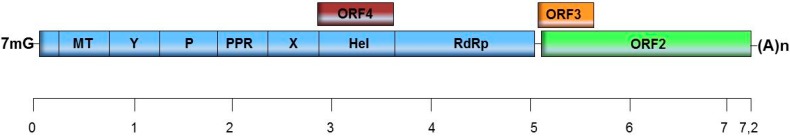
Hepatitis E virus (HEV) genome. The 5′ end of the RNA genome is capped with a 7-methylguanosine (7 mG), and the 3′ end is polyadenylated (poly(A)). Open reading frame 1 (ORF1) encodes nonstructural proteins, including a methyl transferase (MT), cysteine protease (P), helicase (Hel), and RNA polymerase (RdRp), as well as three regions of unknown function (Y domain, polyproline region (PPR), and X domain). ORF4 has only been described in hepatitis E virus genotype 1 (HEV1).

HEV is a single-stranded, positive-sense RNA virus. The 7.2 kb long genome is capped at the 5′ end and polyadenylated at the 3′ end. It contains three open reading frames (ORF), ORF1, ORF2, and ORF3, flanked by noncoding regions (Figure 1). ORF1 encodes a non-structural protein about 1693 amino acids (aa) long, with at least four putative functional domains: methyltransferase, papain-like cysteine protease, helicase, and RNA-dependent RNA polymerase (RdRp). It also has domains that are homologous with those of other plant and animal positive-stranded RNA viruses: the Y domain, the polyproline region (PPR) previously called the hypervariable region, and a macro domain, previously called the X domain [2]. HEV1 was recently shown to have an overlapping reading frame within ORF1, named ORF4. The protein encoded by ORF4 is produced only under condition of endoplasmic reticulum stress and is necessary for the proper functioning of HEV RNA polymerase [3]. ORF2 and ORF3 overlap, and their corresponding proteins are translated from a bicistronic subgenomic RNA. ORF2 encodes the 660 aa capsid protein. Its three domains are S (shell), M (middle) and P (protruding). Lastly, ORF3 encodes a 113 or 114 aa phosphoprotein, depending upon the genotype. In vitro, ORF3 is not required for viral replication, assembly and infection of hepatoma cell line [4], but is required for infection of macaques [5]. This protein is essential for virus egress from infected cells [6]. In serum, HEV particles are associated with lipids and HEV ORF3 protein [7]. Thus, HEV circulates in the blood in a membrane-associated, quasi-enveloped form but is shed into the feces as unenveloped virions [8,9]. HEV particles from serum band at a density of 1.15–1.16 g/mL in a sucrose density gradient, while HEV particles from feces band at 1.27–1.28 g/mL [7]. Interestingly, without prior treatment with detergent, few or no virus particles in both serum and cell culture are captured by anti-ORF2 and anti-ORF3 antibodies [7].

**Figure 2:**
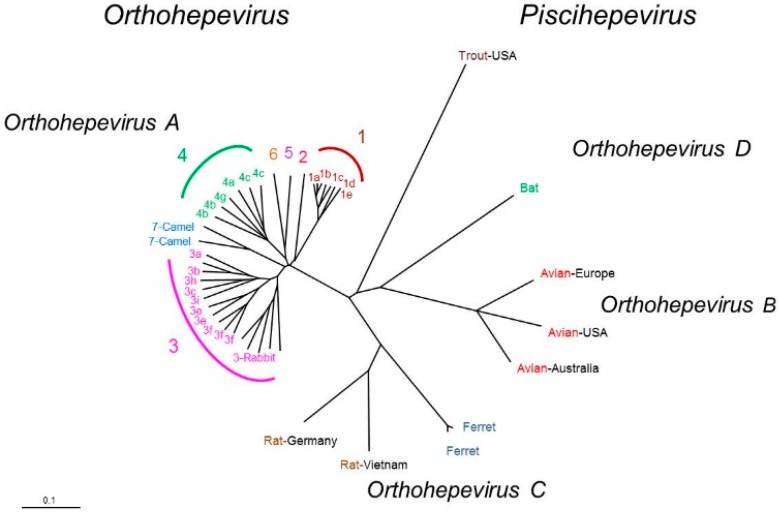
Phylogenetic tree based on full-length sequences of HEV strains. Sequences were aligned using ClustalW (MEGA5) and BioEdit (version 7.0). The phylogenetic tree was created by the neighbour-joining (Kimura two-parameter) method, with a bootstrap of 100 replicates. The species *Orthohepevirus A* includes 7 genotypes (HEV1−7).

The increasing number of HEV strains identified in various hosts has led to a taxonomic scheme that divides the family *Hepeviridae* into two genera: *Piscihepevirus* (cutthroat trout virus) and *Orthohepevirus* (mammalian and avian strains; Figure 2) [10,11]. This last genus has been divided into four species, *Orthohepevirus* *A*, *Orthohepevirus* *B* (infecting birds), *Orthohepevirus* *C* (infecting rodents, soricomorphs, and carnivores), and *Orthohepevirus* *D* (infecting bats). The largest species, *Orthohepevirus* *A*, includes seven genotypes that infect humans (HEV1, 2, 3, 4, & 7), pigs (HEV3 & 4), rabbits (HEV3), wild boar (HEV3, 4, 5, & 6), mongooses (HEV3), deer (HEV3), yaks (HEV4) and camels (HEV7). Among the four major genotypes, HEV1 and HEV2 are restricted to humans and are found in developing countries. HEV3 is widely distributed around the world and HEV4 is found mainly in Asia. The HEV3 and HEV4 genotypes are transmitted zoonotically from pigs, wild boars, deer, and mongooses [12]. Rabbit strains that are closed to HEV3 have recently been described in humans [13]. A liver transplant recipient who had consumed camel meat and milk was found to harbor camel HEV [14]. The transmission of HEV from ferrets, rats, bats, birds, or trout to humans has not yet been demonstrated [12].

## 3. Clinical Course of HEV Infection

### 3.1. Natural History

Hepatitis E causes a self-limiting illness that lasts a few weeks in most patients. Most (>95%) infections are asymptomatic but the disease can be icteric or fulminant. The clinical presentation in developing and developed countries is quite similar [15]. An initial incubation period of 2–6 weeks is followed by symptoms of hepatitis, including fever and nausea, and then by abdominal pain, vomiting, anorexia, malaise, and hepatomegaly. About 60% of patients become jaundiced [16]. Mortality rates during an outbreak can vary from 0.5% to 4.0% of symptomatic infections.

The virus affects mainly young adult males (15–30 years) in developing countries, but pregnant women are particularly vulnerable. The mortality rate may reach 30% during the third trimester of pregnancy [17]. Pregnant women die of obstetric complications such as hemorrhage or eclampsia. Fulminant liver failure can also occur. Stillbirths are common, as is vertical transmission to infants which leads to increased neonatal morbidity and mortality [18]. One study in India found that HEV-related and non-HEV-related acute liver failure during pregnancy had similar mortality rates, although HEV-related acute liver failure was more common during pregnancy [19].

Patients in developed countries who become infected with HEV are usually middle-aged or elderly men (>55 years). Pregnant women do not seem to suffer from severe HEV infections. Patients with underlying liver disease have a poor prognosis in both developing and developed countries [20,21].

HEV3 and HEV4 can persist in immunocompromised patients, including solid organ transplant recipients [22,23,24] and those with human immunodeficiency virus (HIV) infections having a low T CD4+ count (<200/mm^3^) [25,26,27] or hematological disease [28,29,30,31]. To date, there has been no report of patients with an HEV1 infection suffering from chronic hepatitis E. Chronic HEV infection is defined by persistent HEV replication for more than three months [32]. A chronic HEV infection can lead to chronic hepatitis and progress rapidly to cirrhosis [24,33]. Some of these patients may die from decompensated cirrhosis. Retransplantation of liver transplant recipients infected with HEV usually leads to reinfection of the transplanted graft.

### 3.2. Extra-Hepatic Manifestations

Extra-hepatic manifestations can also occur in patients with acute or chronic HEV infection. These include a range of neurological symptoms and impaired kidney function associated with cryoglobulinemia.

Neurological disorders are the most widely documented, with descriptions of Guillain–Barré syndrome, neuralgic amyotrophy, and encephalitis/meningoencephalitis/myositis [34]. In the Netherlands, 5% of patients with Guillain-Barré syndrome have an associated acute HEV infection [35]. Acute hepatitis E was also found in 10% of patients with neuralgic amyotrophy from the United Kingdom and the Netherlands [36]. The pathophysiology of HEV-associated neurological injury remains uncertain. The immune response triggered by an HEV infection may play a role. It seems likely that Guillain-Barré syndrome and neuralgic amyotrophy are immune mediated. Another possibility is that HEV is directly neurotropic; a kidney recipient with a chronic HEV infection was found to have pyramidal syndrome. Analysis of HEV RNA from the cerebrospinal fluid of this patient showed that the variants differed from those in the serum, at the same time, suggesting the presence of neurotropic variants [37], and possibly HEV replication in the central nervous system. A recent study demonstrated that M03.13 oligodendrocytic cells are permissive and support the HEV lifecycle [38]. Human mesodermal and neuroprogenitor cells derived from pluripotent stem cells support HEV replication only when transfected with subgenomic replicon [39].

**Figure 3:**
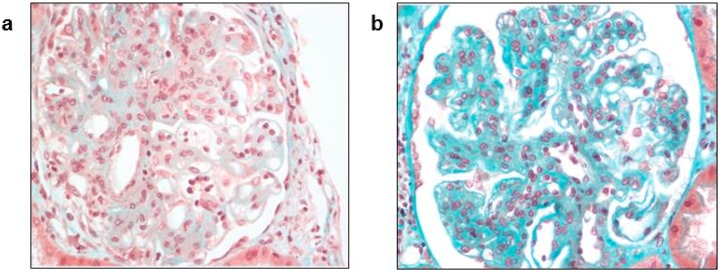
Biopsies of a kidney allograft in a patient with membranoproliferative glomerulonephritis at the acute phase of HEV infection. (**a**) kidney-allograft biopsy at diagnosis; (**b**) kidney-allograft biopsy seven months after HEV clearance (Masson’s trichrome, magnification: × 400).

Kidney injuries and impaired renal function have been reported during acute HEV3 infections, without any clear explanation. Kidney biopsies from patients with biological glomerular abnormalities revealed histological features of membranoproliferative glomerulonephritis (MPGN) (Figure 3), membranous glomerulonephritis and a relapse of immunoglobulin A nephropathy [40]. Cryoglobulins were detected in the serum in most cases, but only one case of HEV-induced cryoglobulinemic crescentic MPGN in an immunocompetent male has been documented [41]. Anti-HEV IgG, anti-HEV IgM, and HEV RNA were all detected in both the serum and the cryoprecipitate. Renal function improved after HEV clearance [41]. The kidney MPGN disease in patients with a hepatitis C virus (HCV) infection is linked to the deposition of immune complexes formed from the HCV antigen, anti-HCV IgG antibodies, and a rheumatoid factor in the glomerulus [42]. A similar mechanism could be at work in HEV infections. Both HEV antigen and RNA were detected recently in the urine of patients chronically infected with HEV [43]. However, there is still no evidence that HEV is directly nephrotoxic or that it can replicate in renal cells.

Lastly, hematological manifestations, such as aplastic anemia and severe thrombocytopenia, can occur. Acute pancreatitis has also been described [15].

## 4. Pathogenesis

The pathogenesis of hepatitis E is poorly understood. Since HEV is presumably transmitted by the fecal-oral route, it is unclear how the virus reaches the liver. Perhaps there is an extra-hepatic site of virus replication. The virus could replicate in the intestinal tract before reaching the liver. Negative strands of HEV RNA, indicating virus replication, have been detected in the small intestine, lymph nodes, colon, and liver of pigs, indicating extra-hepatic HEV replication [44]. HEV then replicates in the cytoplasm of hepatocytes and is released into both blood and bile. The liver damage induced by HEV infection may be immune-mediated by cytotoxic T cells and natural killer (NK) cells since HEV is not cytopathic [45] The virus is shed in the stool [46].

### 4.1. Genetic Susceptibility to HEV

Apolipoprotein E (ApoE) plays an important role in the transport of lipids in the plasma. It may also be involved in the propagation and release of viruses like HCV [47]. Analysis of the proteomes of acutely infected pigs revealed that the ApoE was upregulated, suggesting that it is involved in HEV pathogenesis [48]. ApoE isoforms ε3 and ε4 are significantly associated with protection against HEV infection in American non-Hispanic blacks [49]. Several hypotheses have been formulated to explain this protective role. ApoE could inhibit virus binding by competing with heparan sulfate proteoglycans [50]; ApoE may be in the lipid membrane associated with HEV virions in the blood and could be essential for virus entry [51]; or, lastly, ApoE may modulate the immune response to HEV by regulating T lymphocyte activation and proliferation [52]. It is also possible that this association reflects differences in lifestyle, especially the consumption of pork meat [53].

A (G/A) polymorphism at position 308 in the promotor region of the tumor necrosis factor alpha (TNF-α) is associated with susceptibility to HEV infection. In vitro, the 308A allele results in a seven fold higher TNF-α production [54]. Single nucleotide polymorphisms in the promotor of TNF-α (1031 T/C) and in the promotor of interferon gamma (IFN-γ +874 T/A) seem to contribute to the severity of the disease [55]. The T allele at IFN-γ +874, which is associated with a higher IFN-γ production, was overrepresented in symptomatic cases [55].

### 4.2. Innate Immune Response

Microarray analyses of the intrahepatic transcriptome in serial liver biopsies obtained from chimpanzees infected with HEV or HCV suggest that HEV is more susceptible than HCV to the innate immunity induced by interferon alpha (IFN-α) [56]. However, HEV has developed mechanisms to suppress IFN-α signaling. In vitro studies on A549 human lung epithelial cells [57] and Huh7 hepatocarcinoma cells [58] indicate that IFN-induced phosphorylation of signal transducer and activator of transcription STAT1 can be inhibited by the ORF3 protein, leading to a down regulation of two key antiviral proteins, dsRNA-activated protein kinase and 2′,5′-oligoadenylate synthetase. Nan et al., working on HEK293T cells, showed that ORF3 protein enhanced type I IFN production by interacting directly with the pattern recognition receptor (PRR) retinoic acid-inducible gene I (RIG-I) [59]. In the same cells, they found that ORF1 protein inhibited RIG-I signaling and prevented interferon beta (IFN-β) induction by de-ubiquitination of RIG-I and tank binding kinase 1 [60]. The inhibitory effect seems to be rather small, making only a minor contribution to HEV’s resistance to IFN. However, the gene silencing of the key component of the Janus kinase (JAK)-STAT cascade of the IFN signaling, including JAK1, STAT1, and interferon regulatory factor 9, stimulated HEV infection/replication, indicating that the IFN cascade can restrict HEV infection [58].

Analyses of gene/protein expression in A549 cells infected with HEV showed a robust induction of inflammatory cytokines/chemokines, such as interleukin (IL)-6, IL-8, TNF-α and RANTES (regulated on activation, normal T cell expressed and secreted). HEV infection also led to the activation of both nuclear factor kappa-light-chain-enhancer of activated B cells (NF-κB) and IFN regulatory factor 3 (IRF3), two transcription factors activated in innate immune signaling pathways [61].

NK and natural killer T (NKT) cells could also play a major role in the innate immune response to HEV. The proportion of CD4+ cells in the peripheral blood of patients with acute hepatitis E is greater than in the blood of healthy controls, but that of CD8+ cells is unchanged. In vitro stimulation with ORF2 protein do not show expansion of HEV ORF2-specific CD4+/CD69+ cells producing helper T cell type 1 (IFN-γ and TNF-α) or helper T cell type 2 (IL-4) cytokines. Conversely, peripheral blood mononuclear cells (PBMCs) from patients acutely infected with HEV have higher IFN-γ concentrations than do PBMCs from controls when stimulated in vitro with ORF2 protein. Thus, CD4+ cells producing IFN-γ could be NKT [62]. The proportions and activation status of the peripheral NK and NKT cells are reversibly altered during acute hepatitis E, with fewer NK (CD3−/CD56+) and NKT (CD3+/CD56+) cells among PBMCs [63]. However, the proportion of activated NK cells in patients with acute hepatitis E is much greater than in healthy controls. The apparent depletion of total NK and NKT cells among PBMCs could reflect the preferential accumulation of these cells in the liver of infected patients [63]. An immunohistological study of liver biopsies from HEV-infected acute liver failure patients showed that their counts of CD56+ cells were significantly higher than in biopsies from patients infected with HAV, HBV or HCV [64].

### 4.3. Addaptive Response

**Figure 4:**
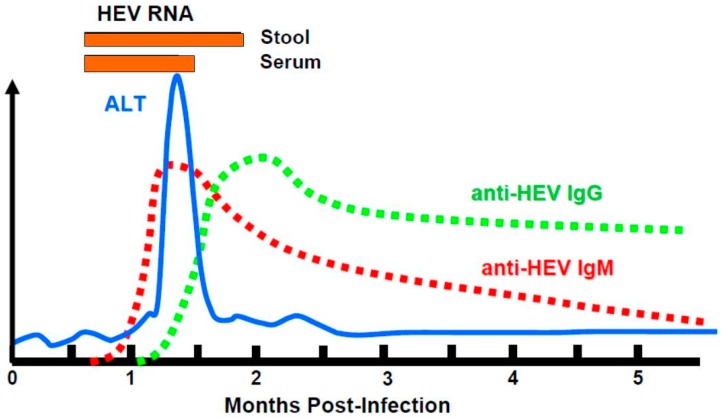
Course of an acute HEV infection.

A serological anti-HEV response is generally detected in patients at the time of onset of illness. Anti-HEV IgMs are detected in the early phase of clinical illness, and can persist for several months. Anti-HEV IgG appears shortly after the IgM response and can last several years (Figure 4). Cross protection is possible due to the existence of only one serotype [65]. The capsid protein contains several neutralizing epitopes [66]. Anti-HEV antibodies can also be induced by vaccination. The only available vaccine, Hecolin^®^ (Xiamen Innovax Biotech, Xiamen, China), is composed of a truncated HEV capsid protein, p239, that confers protection against hepatitis E infection for up to 4.5 years [67,68].

The risk of HEV reinfection is unclear. Studies on humans and primates indicate that anti-HEV IgG antibodies are protective [69,70,71]. Although the minimum protective concentration of antibodies has not been determined, a vaccine study suggests that an antibody concentration of 2.5 World Health Organization (WHO) units/mL is protective [68]. Solid organ transplant recipients can be reinfected when the antibody concentration is below 7 WHO units/mL [72].

Several studies have provided evidence that effector T cells are activated during acute hepatitis E, with infected patients having more CD8+ cells than healthy controls [73]. The proportions of PBMCs producing IFN-γ in response to stimulation with recombinant ORF2 or ORF3 proteins were also higher in patients than in healthy controls [73]. Using immunohistochemistry in liver biopsies from patients with HEV-induced acute liver failure, Prabhu et al. demonstrated the infiltration of activated CD8+ T cells in liver [64].

An increased expression of CD11a integrin in naïve CD45RA+ T cells, as well as overexpression of CCR5 and CCR9, was also reported. The presence of an expanded CD45RA+ CD11a high subpopulation during the early phase of acute infection suggests enhanced recruitment of these cells from the periphery to the target tissue. Thus, CD45RA+ CD11a high CCR5+ cells are involved in the pathogenesis of HEV infection [74]. Patients with acute hepatitis E were found to have a greater proportion of CD4+ CD25+ forkhead box P3+ (FoxP3+) regulatory T cells and higher concentrations of IL-10, a signature cytokine of regulatory T cells, than healthy controls. This suggests that these cells are important in the acute phase of the disease, although their exact role needs further investigation [75]. Lastly, specific T cell immunity seems also to confer a cross-protection against HEV1 and HEV3 [76], which could protect patients exposed to genotype 3 when they travel to areas where HEV1 is endemic.

## 5. Pathogenesis of Fulminant Hepatitis

Fulminant hepatitis is most likely to occur in men and women suffering from chronic liver disease [77] and in pregnant women [17].

### 5.1. Fulminant Hepatitis E in the General Population

The reasons why a hepatitis E infection becomes fulminant are still obscure. Viral factors may be important, as suggested by the observation that HEV4 infections tend to be more severe than those of other strains [78]. However, a recent study re-examined the published evidence for an association between fulminant hepatic failure (FHF) and HEV genotypes and concluded that host factors rather than virus genotype, variants, or specific aa substitutions are responsible for the development of fulminant hepatitis [79].

There is little agreement about the mechanism underlying FHF. Saravanabalaji et al. reported that stimulating both Th1 and Th2 type immune responses could play a role in liver failure. Patients with FHF were found to have higher anti-HEV IgM and IgG titers than those with self-limiting infections [80], and PBMCs from patients with FHF were found to produce higher IFN-γ, TNF-α, IL-2, and IL-10 concentrations after stimulation with ORF2 peptides than PBMCs from controls. In contrast, Srivastava et al. reported less marked antiviral cellular immune responses and heightened humoral antiviral responses in patients with fulminant hepatitis E than in patients with uncomplicated infection and control patients [81]. The heightened humoral response was associated with a more severe HEV disease in both studies.

The situation in peripheral blood may not reflect what occurs at the site of infection. CD4+ T cells are more frequent in the livers of patients with fulminant hepatic failure caused by an HEV infection [64] and CD8+ T cells have been shown to infiltrate the liver of patients with fulminant hepatitis E [64,82]. Thus, cytotoxic CD8+ T cells could be particularly important in the pathogenesis of fulminant hepatitis.

### 5.2. Immunopathogenesis in Pregnant Women

The cause of elevated maternal mortality (30%, with most deaths occurring in the third trimester) of pregnant women infected with HEV1 living in developing countries has been the subject of many studies, but it is still unclear. HEV genotype could explain, at least in part, the poorer outcome in pregnant women since HEV3 is not particularly deadly for pregnant women [83,84]. No data are available for HEV4 infection during pregnancy.

Pregnancy is associated with changes in sex hormone levels and the immune system that are designed to protect the fetus from the maternal immune system. A shift from a Th1-dominated immune response to a Th-2 dominated one,“Th2 bias”, may help protect the fetus by suppressing macrophage activation [85]. Pal et al. confirmed the existence of a Th2 bias in pregnant women infected with HEV, but its implication for the severity of a hepatitis E infection is unknown [86]. Women with acute liver failure (ALF) presented a reduced expression of toll-like receptor (TLR) 3/TLR7/TLR9, a type of PRR that plays a key role in the innate immune system, and have weaker phagocytic macrophages than women with acute viral hepatitis E [87]. However, the phagocytic capacity of the monocytes of the two groups was essentially the same. Impaired monocyte-macrophage function in pregnant women with ALF could contribute to an inadequate innate immune response, and hence to the development and severity of ALF. Kumar et al. reported that high concentrations of cytokines (TNF-α, IL-6, IFN-γ and TGF-β1) may also be associated with an adverse pregnancy outcome [88].

An increased incidence of FHF was reported in pregnant women with the progesterone receptor gene mutations PROGINS haplotype [89]. PROGINS carriers with HEV infection showed reduced expression of progesterone receptor and progesterone-induced blocking factor (PIBF). PIBF exerts its anti-abortive activity by inhibiting NK cells and influencing both the humoral and cellular immune responses [90,91]. Other host factors such as nutritional status or differences in major histocompatibility complex may also influence the immune response of pregnant women to an HEV infection [92]. This may be why an HEV infection is benign in pregnant women in Egypt although it is caused by HEV1 [17].

Lastly, pregnancy-associated hormones can also contribute to a poor outcome. The concentrations of estrogen, progesterone, and β-human chorionic gonadotrophin in HEV-positive pregnant FHF women are higher than in HEV-negative pregnant FHF women or controls [93]. An in vitro study showed that serum from pregnant women, especially those in the third trimester, enhanced the replication of HEV by inhibiting estrogen receptor and type I IFN expression [94]. While some studies have found that the high HEV RNA concentrations in HEV-infected pregnant women were associated with a poor outcome [89,95]; another study by Saravanabalaji et. al, did not confirm these results, with only 1/14 pregnant women having detectable HEV RNA [80].

## 6. Pathogenesis of Chronic Infection in Immunocompromised Patients

Most studies of the pathogenesis of the chronic HEV installation have involved solid organ transplant (SOT) recipients. The incidence of HEV infection in these patients varies from 0.9% to 3.5%, based on the detection of HEV RNA, and acute infections become chronic in nearly 60% of them [96]. 

The use of tacrolimus rather than cyclosporin was found to be associated with HEV persistence in SOT patients [96]. Both cyclosporin and tacrolimus are immunosuppressive; they inhibit the calcineurin phosphatase in lymphocytes. However, tacrolimus is more potent than cyclosporin. It impairs the specific T cell response to HEV more efficiently [97]. In vitro studies have shown that both of these calcineurin inhibitors promote HEV replication by inhibiting cyclophilins A and B, while mycophenolic acid, an inhibitor of inosine 5′ monophosphate dehydrogenase, inhibits HEV replication [98]. Rapamycin and everolimus also promote HEV replication in vitro by inhibiting mechanistic target of rapamycin (mTOR), showing that the PI3K-PKB-mTOR pathway acts as a cell restriction factor [99]. Patients given mTOR inhibitors have higher plasma concentrations of HEV RNA, but mycophenolic acid does not influence HEV replication in vivo. A low platelet count is also associated with HEV persistence [96]. Platelet depletion reduced the accumulation of virus-specific cytotoxic T lymphocytes in the livers of transgenic mice infected with hepatitis B virus. Consequently, organ damage is also reduced [100]. 

The host immune response may also contribute to the development of a persistent infection. The response of the IFN-stimulated genes (ISG) of renal transplant recipients who did not clear their HEV infection was higher than that of the ISG response of patients who cleared their HEV [101]. This suggests that activation of the interferon system does not lead to spontaneous HEV clearance. The increased expression of ISG in patients with a chronic HEV infection seems to favor virus persistence by causing the interferon signaling pathway to become refractory. Lower concentrations of IL-1Ra and soluble IL-2R, together with higher concentrations of chemokines during the acute phase, are also associated with HEV persistence [102]. 

An HEV infection is likely to become chronic in profoundly immunosuppressed patients. The CD2+, CD3+, and CD4+ T-cell subsets are significantly lower in these patients than in those who spontaneously clear the virus [22]. Chronic HEV infections are more frequent in patients who are also infected with HIV and have a low CD4+ T cell count [25,26,27]. In addition, the HEV-specific T cell proliferative responses of SOT patients, particularly those with a chronic infection, are decreased. The development of HEV-specific IFN-γ-producing cells seems to be associated with a favorable outcome in this population [103,104]. A recent study has shown that the gamma delta T cells (γδ T) cells of SOT patients are mobilized during the acute phase of infection [105]. Immunocompetent patients do not produce this immune response, suggesting that SOT patients mobilize a larger fraction of their immunity due to immunosuppressive therapy. The role of these cells at the acute phase of the infection needs further investigation. 

Virus factors may also contribute to the persistence of an HEV infection. Greater quasispecies heterogeneity in ORF1 and ORF2 regions during the acute phase of infection is associated with HEV persistence [102,106]. The Ka/Ks ratio, an indirect indicator of the selection pressure on a quasispecies, in the M domain of the virus capsid protein is also lower in patients developing a chronic HEV infection than in patients who have cleared HEV spontaneously [102]. The M domain contains T cell epitopes, highlighting the importance of the cellular immune response for HEV clearance. The Ka/Ks ratio of the virus domains containing B cell epitopes in the two groups of patients were not different [106].

**Figure 5:**
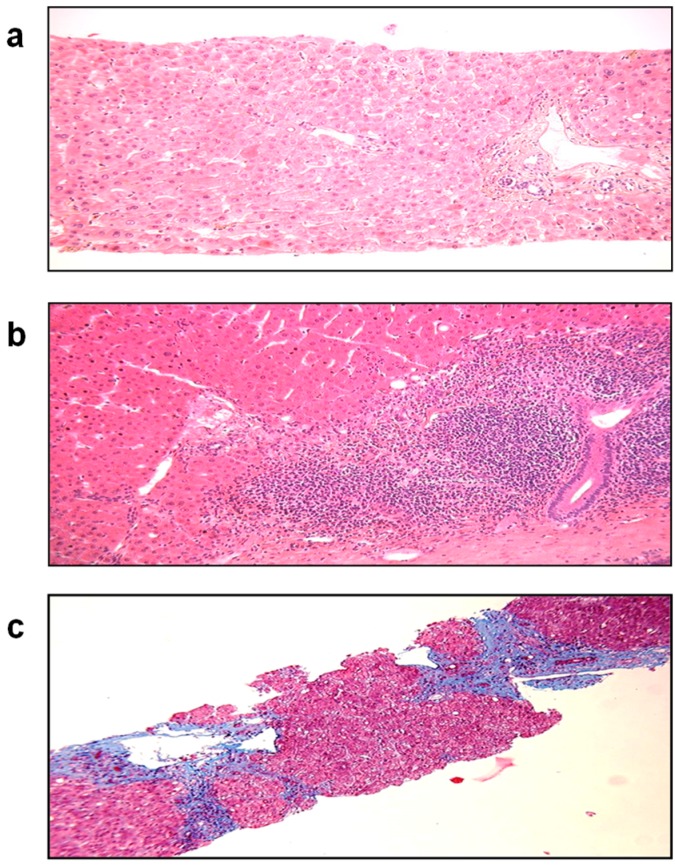
Histological patterns of liver biopsies taken from a patient with a chronic HEV infection. (**a**) initial liver biopsy; (**b**) inflammation after 15 months of chronic HEV infection; (**c**) cirrhosis after 38 months of chronic HEV infection. (Masson’s trichrome, magnification × 100).

Nearly 10% of SOT patients with HEV develop cirrhosis within 3–5 years following the primary infection (Figure 5). The slow diversification of the P capsid domain seems to be associated with progression to liver fibrosis [102]. This could indicate that more aggressive variants are selected in fibrosers; however, additional large study findings are still needed to support this idea.

Lastly, chronically infected patients have recently been found to harbor recombinant HEV-host variants in [107,108,109]. The PPR regions of these recombinant variants were found to include fragments of human genes of varying origin (ribosomal genes S17 or S19, inter alpha trypsin inhibitor). Such recombinant variants had a replicative advantage in vitro. Duplications and insertions of the HEV genome were also detected [107,110]. Their influence on HEV infection is unknown and needs further investigation.

## 7. Animal and in vitro Models

### 7.1. Animal Models

Non-human primates, including *Rhesus* and *Cynomolgus* macaques and chimpanzees, are all primary models for studying the clinical course of infections by the four major genotypes of HEV. However, most of these studies in non-human primates have used intravenous inoculation with HEV because oral inoculation requires much higher doses of virus [46]. 

Several animal species are naturally susceptible to HEV3 and HEV4. The first non-human strain of HEV was found in pigs [111]. Pigs have been very useful for studying HEV cross-species infections, replication, and pathogenesis [44,112]. They can be used to study chronic HEV infections when co-infected with porcine reproductive and respiratory syndrome virus [113]. 

Rabbits also provide an interesting model for studying HEV pathogenesis. The rabbit strain of HEV can infect both pigs and macaques, while, conversely, rabbits can be infected with HEV4 [114,115]. Another advantage is that rabbits can be used to simulate the high mortality rate associated with pregnancy in humans [116]. Rabbits can even be used as a model of chronic infection with HEV3 and extra-hepatic manifestations [117].

Ferrets are also a potentially interesting species as they support persistent infections with ferret HEV [118]. Whether ferrets are susceptible to infection with other zoonotic strains remains to be seen. Mongolian gerbils were also successfully infected with swine HEV [119]. Rats are not a very suitable model for human HEV infection, since HEV1, HEV2, HEV3, HEV4 strains do not infect rats [120,121]. Conversely, the rat HEV strain does not infect *Rhesus* monkeys [121]. Mice with humanized livers have recently been established for studying chronic HEV infections [122,123,124]. They seem to be a valuable tool for exploring HEV replication and evaluating the efficacy of antiviral molecules.

Lastly, chickens have been used to identify extra-hepatic sites of avian HEV replication [125]. The impact of deletion of the PPR on HEV infectivity was also studied with avian HEV strains [126,127].

### 7.2. Cell Culture Systems for HEV Replication

HEV3 extracted from the feces of a patient with an acute HEV infection has been grown on PLC/PRF/5 (hepatoma) and A549 (lung adenocarcinoma) cells. This initial propagation of an HEV strain was successful because of the high HEV titer in the inoculum (2.0 x 10^7^ copies/mL) [128]. Another fecal suspension of HEV4, this time from a Japanese patient with fulminant hepatitis E, was used to establish a culture system on PLC/PRF/5 and A549 cells [129]. More recently, the HEV3 Kernow strain purified from the feces of a chronically infected patient was also replicated efficiently on HepG2/C3A (hepatoma) cells [108]. Adaptation of the Kernow strain to in vitro growth selected a recombinant virus containing an insertion of 174 ribonucleotides (58 amino acids) from a gene encoding the human S17 ribosomal protein. Other strains with insertions of human S19 ribosomal protein (117 nt) or human inter-α trypsin inhibitor (75 nt) have also been grown efficiently on HepG2C3A cells [106,109]. HEV1, HEV3, and HEV4 strains from serum samples can also replicate efficiently in PLC/PRF/5 and A549 cells. Replication depends on the HEV RNA concentration in the inoculum, but is not influenced by any anti-HEV antibodies in serum [7]. It is important to distinguish between viruses obtained from the feces and serum because the presence of lipid modifies the mechanism by which virus enters the cell [130].

Cells of the human hepatoma-derived cell line HepaRG and the porcine embryonic stem cell-derived cell line PCM19 have been found to support the replication of an HEV3 strain isolated from swine feces. The morphological and functional properties of these two cell lines are similar to those of primary hepatocytes [131]. Lastly, hepatocytes derived from pluripotent stem cells have been shown to support the complete replication cycle of HEV. This is an attractive in vitro model system (non-cancerous hepatocytes) for studying HEV replication [39].

## 8. Conclusions

The pathogenesis of HEV infection involves a complex interplay between the virus and its host, particularly the host immune system. The currently recognized clinical phenotype of HEV is primarily hepatological but the range and incidence of HEV-associated clinical symptoms has become considerably broader over the past few years. The new animal models and in vitro systems will play a significant part in improving our understanding of HEV pathogenesis.
